# ^1^H, ^15^N, ^13^C backbone resonance assignments of human soluble catechol *O*-methyltransferase in complex with *S*-adenosyl-l-methionine and 3,5-dinitrocatechol

**DOI:** 10.1007/s12104-016-9720-9

**Published:** 2016-12-15

**Authors:** Sylwia Czarnota, Nicola J. Baxter, Matthew J. Cliff, Jonathan P. Waltho, Nigel S. Scrutton, Sam Hay

**Affiliations:** 1 Manchester Institute of Biotechnology and School of Chemistry, The University of Manchester, 131 Princess Street, Manchester, M1 7DN UK; 2 Department of Molecular Biology and Biotechnology, Krebs Institute for Biomolecular Research, The University of Sheffield, Firth Court, Western Bank, Sheffield, S10 2TN UK

**Keywords:** Enzyme, *S*-adenosyl-l-methionine, Backbone resonance assignment, Transverse relaxation optimized spectroscopy, Triple-labelled Protein

## Abstract

Catechol *O*-methyltransferase (COMT) is an enzyme that plays a major role in catechol neurotransmitter deactivation. Inhibition of COMT can increase neurotransmitter levels, which provides a means of treatment for Parkinson’s disease, schizophrenia and depression. COMT exists as two isozymes: a soluble cytoplasmic form (S-COMT), expressed in the liver and kidneys and a membrane-bound form (MB-COMT), found mostly in the brain. Here we report the backbone ^1^H, ^15^N and ^13^C chemical shift assignments of S-COMT in complex with *S*-adenosyl-l-methionine, 3,5-dinitrocatechol and Mg^2+^. Assignments were obtained by heteronuclear multidimensional NMR spectroscopy. In total, 97 % of all backbone resonances were assigned in the complex, with 205 out of a possible 215 residues assigned in the ^1^H-^15^N TROSY spectrum. Prediction of solution secondary structure from a chemical shift analysis using the TALOS+ webserver is in good agreement with published X-ray crystal structures.

## Biological context

Catechol *O*-methyltransferase (COMT, EC 2.1.1.6) is a ubiquitous bisubstrate magnesium-dependent enzyme found in plants, animals and microorganisms. It catalyses the transfer of a methyl group from *S*-adenosyl-l-methionine (SAM) to one of the hydroxyl oxygen atoms (preferentially the 3-hydroxyl) in a catechol substrate (Mannisto and Kaakkola 1999). Physiological substrates of COMT are catecholamine neurotransmitters such as dopamine, noradrenaline, adrenaline and their metabolites. COMT also methylates catecholic steroids such as 2-hydroxyestradiol as well as a range of other catecholic compounds including neuroactive drugs such as l-dopa, α-methyldopa and isoproterenol (Ball et al. 1972; Borchardt 1980; Guldberg and Marsden 1975). COMT inhibition is a means of treating Parkinson’s disease, schizophrenia and depression (Fava et al. 1999; Harrison et al. 2015; Kiss and Soares-da-Silva 2014). There are two isoforms of human COMT: soluble cytoplasmic COMT (S-COMT), which is mainly intracellular, and a membrane-bound form (MB-COMT), which has a single-span helix contained within a 50 amino acid extension at the N-terminus. Genetic studies have demonstrated that both soluble and membrane-bound isoforms of human COMT are coded by a single gene, using two separate promoters, assigned to chromosome 22q11.2 (Tenhunen et al. 1994). However, S-COMT possesses *K*_m_ values for catecholamines that are ~15 times higher than those reported for MB-COMT, and in addition has a lower affinity for the SAM cofactor (Lotta et al. 1995). Several X-ray crystal structures have been solved for COMT enzymes from a range of organisms together with different substrate/inhibitor complexes. S-COMT has a single domain α/β-folded structure with eight α-helices and seven β-strands. The N-terminal region is composed of three α-helices, the central section has five α-helices arranged around a five-stranded parallel β-sheet, while the C-terminal part consists of two antiparallel β-strands. The active site is located on the outer surface of the enzyme and includes a SAM binding pocket and a substrate binding site situated in the vicinity of a bound catalytic Mg^2+^ ion (Vidgren et al. 1994).

## Methods and experiments

### Protein expression and purification

The human S-COMT construct used in this study has a 12 residue hexa-histidine tag and cloning sequence positioned at the N-terminus (MHHHHHHENLYFQG…). The canonical S-COMT sequence begins at Q1 and here the canonical S-COMT numbering system is used throughout. This construct also contains valine (rather than methionine) at the allelic polymorphism position located at residue 108 of S-COMT (Lachman et al. 1996; Rutherford et al. 2008a). A synthetic, codon-optimised human S-COMT gene cloned into a pEX-A2 plasmid was purchased from Eurofins Genomics. This gene was then cloned into a pET21a plasmid for expression purposes. ^2^H,^15^N,^13^C-labelled protein was expressed using *Escherichia coli* strain BL21(DE3) (Stratagene) in a defined isotopically labelled minimal media, following the protocol of Reed et al. (2003). The cells were grown at 37 °C with shaking until A_600 nm_ reached 0.6–0.8, were cooled to 30 °C and induced by adding isopropyl-β-d-thiogalactopyranoside (IPTG) to a final concentration of 0.4 mM. Cells were harvested 12–14 h after induction using centrifugation at ~6000 rpm for 20 min at 4 °C. The cell pellet was collected and stored at −20 °C until further use. All purification steps were performed at 4 °C. Harvested cells were allowed to thaw and were then resuspended in cell lysis buffer comprising 50 mM sodium phosphate, 300 mM NaCl, 10 mM imidazole, pH 7.4 and containing cOmplete™, Mini, EDTA-free protease inhibitor tablets (Roche) (one tablet per 50 mL of buffer), 10 µg/mL DNase and 10 mM MgCl_2_. Cells at a concentration of ~100 mg of cell pellet/mL were lysed on ice by sonication for 15 cycles of pulsation for 15 s with 45 s intervals. The cell extract was then separated by ultracentrifugation at ~48,000×*g* (20,000 rpm) for 30 min at 4 °C in a Beckman Coulter J26-XP Avanti centrifuge using rotor JA 25.50. The supernatant was then filtered using 0.45 μm syringe filters (Sartorius Mechatronics UK Ltd) before loading onto a 5 mL His-Trap FF affinity Ni-Sepharose column (GE Healthcare) connected to an AKTA purification system (GE Healthcare) that had been previously washed with at least five column volumes of filtered and degassed water and equilibrated by washing with ten column volumes of filtered and degassed cell lysis buffer. Proteins bound to the Ni resin were eluted with a gradient of 10–300 mM imidazole in cell lysis buffer over 16 column volumes or with a two-step elution comprising eight column volumes of 10 mM imidazole in cell lysis buffer and then eight column volumes of 300 mM imidazole in cell lysis buffer. Fractions showing S-COMT content, observed as a peak in UV absorbance at 280 nm, were pooled, checked for purity by SDS–PAGE, concentrated by VivaSpin (10,000 MWCO, GE Healthcare) and loaded onto a Superdex 75 26/60 size exclusion column (Fisher Scientific) connected to an AKTA purification system. The column was washed with at least 1.5 column volumes of filtered and degassed water and equilibrated with two column volumes of filtered and degassed gel filtration buffer (50 mM Tris–HCl buffer pH 7.5 containing 50 mM NaCl and 10 mM DTT) prior to use. S-COMT was eluted with two column volumes of gel filtration buffer, and then checked for purity by SDS–PAGE. Chromatograms (280 nm detection) monitoring the size exclusion purification show two separated peaks, which correspond to monomeric and dimeric S-COMT as confirmed by native gel electrophoresis and mass spectrometry (not shown). The fraction of monomer was typically 40–80 % of total purified S-COMT. The monomeric form of the protein was used for further investigations. Once purified, back exchange to amide protium atoms in perdeuterated S-COMT was promoted by overnight incubation in 50 mM Tris–HCl, 10 mM DTT, pH 9.0 at 25 °C, followed by VivaSpin-mediated buffer exchange at 4 °C into 50 mM Tris–HCl, 10 mM DTT, 50 mM NaCl, pH 7.5. Protein concentrations were estimated by absorbance at 280 nm measured with a NanoDrop (ε_280_ = 24,785 M^−1^ cm^−1^) and Bio-Rad protein concentration assays, following the manufacturers’ protocols. NMR experiments were performed on S-COMT samples obtained within 1 day of purification. The stable isotopically-labelled compounds ^15^NH_4_Cl (99 %), ^13^C_6_,^2^H_7_-d-Glucose (U-^13^C_6_, 99 %; 1,2,3,4,5,6,6-d_7_ 97–98 %) and ^2^H_2_O (99.8 %) were purchased from Goss Scientific. All other reagents, including S-adenosyl-l-methionine (SAM) and 3,5-dinitrocatechol (DNC) were purchased with the highest purity available from Sigma-Aldrich (Dorset, UK) and used as received.

### NMR experiments

All NMR measurements were performed at 298 K, using standard pulse sequences on an 800 MHz Bruker Avance III NMR spectrometer fitted with a TCI cryoprobe equipped with Z gradients and TopSpin software version 3.2 housed in the Manchester Institute of Biotechnology. NMR samples containing 0.5 mM ^2^H,^15^N,^13^C-labelled human S-COMT, 5 mM SAM, 5 mM DNC and 2.5 mM MgCl_2_ in 50 mM Tris–HCl buffer, 50 mM NaCl, 10 mM DTT, 2 mM NaN_3_, pH 7.5 were loaded into 5-mm diameter NMR tubes. ^2^H_2_O was added to the protein samples (10 % v/v) to allow a deuterium lock and 0.5 % v/v trimethylsilyl propanoic acid (TSP) was added as a reference signal. ^1^H chemical shifts were referenced relative to the internal TSP signal, whereas ^15^N and ^13^C chemical shifts were indirectly referenced using nuclei-specific gyromagnetic ratios. For the backbone ^1^H, ^15^N and ^13^C resonance assignment, standard Bruker ^1^H-^15^N TROSY and TROSY-based 3D HNCA, HNCACB, HN(CO)CACB, HN(CA)CO and HNCO spectra were acquired using non-uniform sampling with a multidimensional Poisson Gap scheduling strategy with sinebell weighting (Hyberts et al. 2013). A 30 Hz (0.4 ppm) resolution in the carbon dimension was obtained after processing. The HNCO spectrum, with one peak per residue in the carbon dimension was obtained with 230 hypercomplex points, whereas spectra with two peaks per residue (HNCA, HN(CO)CACB, HN(CA)CO) were obtained with 460 hypercomplex points and the HNCACB spectrum with four peaks per residue was obtained with 920 hypercomplex points.

### Resonance assignments and data deposition

Backbone ^1^H_N_, ^15^N, ^13^C_α_, ^13^C_β_ and ^13^C’ chemical shifts were assigned for S-COMT in the S-COMT:SAM:DNC:Mg^2+^ complex using standard triple resonance methodology (Gardner and Kay 1998). Spectra were processed with TopSpin software version 3.2. Peak picking and frequency matching was performed within CCPNMR Analysis version 2.4 (Vranken et al. 2005) and the backbone assignments were checked independently using a simulated annealing algorithm employed by the “asstools” assignment program (Reed et al. 2003). The backbone ^1^H, ^15^N and ^13^C chemical shifts have been deposited in the BioMagResBank (http://www.bmrb.wisc.edu/) under the BMRB accession code 26848.

**Fig. 1:**
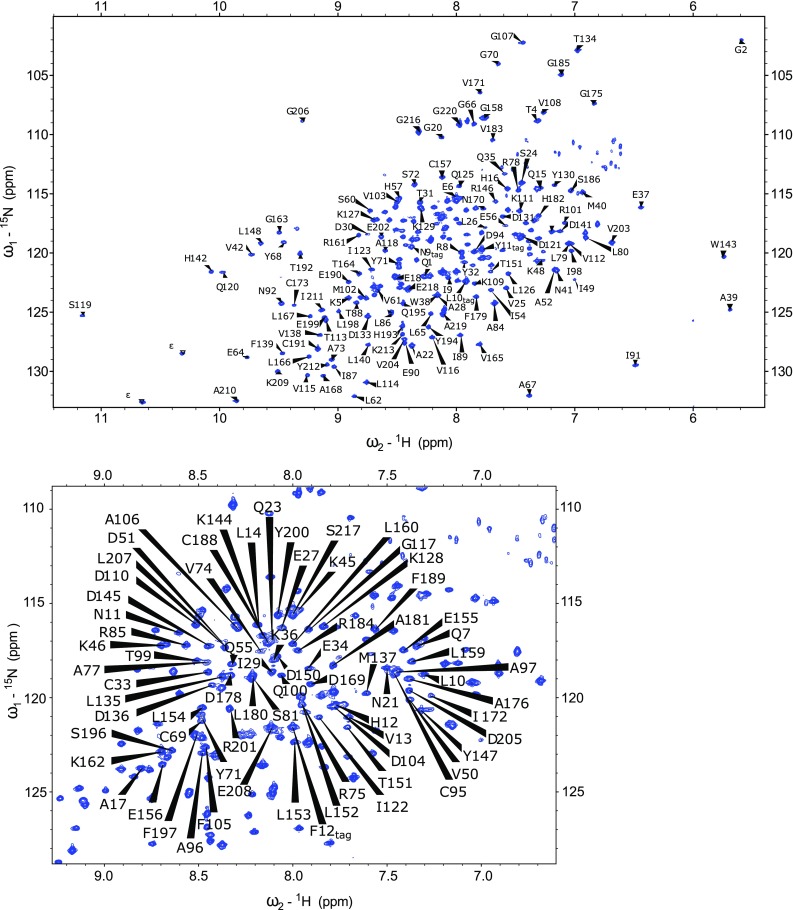
2D ^1^H-^15^N TROSY spectrum of the human S-COMT:SAM:DNC:Mg^2+^ complex recorded at pH 7.5 and 298 K. The assignments of backbone amide resonances are indicated by residue type and sequence number, following the canonical S-COMT nomenclature. Residues of the cloning sequence are referred to by their residue type and position in the cloning tag together with a tag indicator (e.g. N9_tag_)

Excluding the ten proline residues and the first eight residues of the N-terminal cloning tag from the 233-residue S-COMT protein sequence, 205 out of a total of 215 residues were assigned in the ^1^H-^15^N TROSY spectrum of the S-COMT:SAM:DNC:Mg^2+^ complex (Fig. 1). In total, 97 % of all backbone resonances were assigned (95 % of ^1^H_N_, 95 % of ^15^N, 98 % of ^13^C_α_, 97 % of ^13^C_β_ and 98 % of ^13^C’ nuclei). There is evidence for exchange dynamics occurring on a slow NMR timescale due to the presence of duplicate spin systems in the ^1^H-^15^N TROSY and 3D correlation spectra. *Cis–trans* proline isomerisation at P221 is the most likely source of conformational dynamics responsible for spin system duplication at A219 and G220. There is also spin system duplication for Q1 and G2, where the cloning tag meets the S-COMT sequence.

There are ten residues that remain unassigned in the ^1^H-^15^N TROSY spectrum (D3, G43, D44, G47, V53, Q58, M76, G83, S187 and G214). From the crystal structure (PDB: 3BWM; (Rutherford et al. 2008b), Fig. 2), D3, Q58, G83, S187 and G214 are located at the surface of the protein, mostly in solvent exposed loops, and as a consequence the ^1^H-^15^N TROSY correlations are likely to be attenuated beyond detection by fast exchange with solvent. Several residues in the third α-helix (G43-Q58) and fourth α-helix (G70-R78) have ^1^H-^15^N TROSY peak intensities that are broadened by conformational exchange; specifically these are: K48, I49, D51, I54, E56, V74, R75 and A77. Such exchange behaviour points to dynamics occurring on the millisecond timescale in this region of the protein, which are the likely source of the broadening beyond detection of the ^1^H-^15^N TROSY correlations of G43, D44, G47, V53 and M76. An overlay of S-COMT crystal structures (PDB: 4PYI, 3A7E, 3BWM, 4PYQ, 4P7J) shows that the last turn of the second α-helix (C33-K36) and the first turn of the third α-helix (G43-K46) has positional heterogeneity resulting from the active site loop (E37-V42) occupying alternative conformations. One consequence of these conformational differences requires that the sidechain donors of R75 coordinate the sidechain acceptors of D44 and D51 differently, which might account for the exchange broadening behaviour observed for these residues, together with residues in their immediate vicinity.

**Fig. 2:**
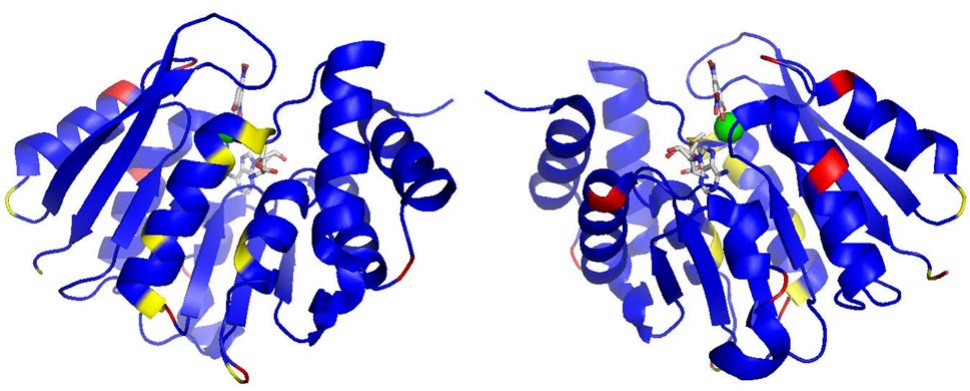
Two orthogonal representations of the backbone assignments mapped onto the X-ray crystal structure of the human S-COMT:SAM:DNC:Mg^2+^ complex (PDB: 3BWM; (Rutherford et al. 2008b)). Assigned residues are coloured *blue*, proline residues are coloured *red*, and all unassigned residues are coloured *yellow*. SAM and DNC are shown as CPK-coloured sticks and the magnesium ion is indicated as a *green sphere*

**Fig. 3:**
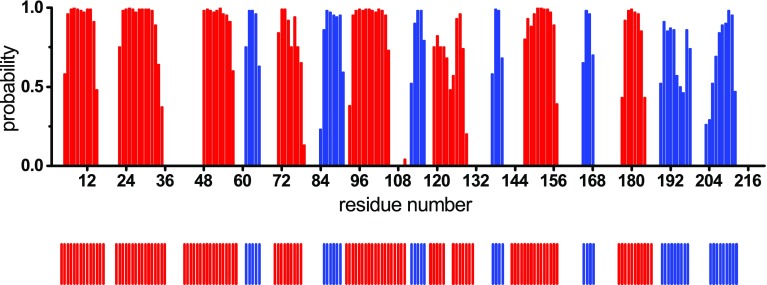
Backbone secondary structure prediction of S-COMT in the S-COMT:SAM:DNC:Mg^2+^ complex obtained with TALOS+ (Shen et al. 2009) using the backbone ^1^H_N_, ^15^N, ^13^C_α_, ^13^C_β_ and ^13^C’ chemical shifts. The secondary structure prediction is shown as *red bars* for α-helices and *blue bars* for β-strands, with the height of the bars representing the probability of the secondary structure assigned by the software. The secondary structure derived from the X-ray crystal structure of the human S-COMT:SAM:DNC:Mg^2+^ complex (PDB: 3BWM; (Rutherford et al. 2008b)) is reported below the *panel* in the *same colour* representation

The secondary structure content of S-COMT was predicted by uploading the backbone ^1^H_N_, ^15^N, ^13^C_α_, ^13^C_β_ and ^13^C’ chemical shifts of the S-COMT:SAM:DNC:Mg^2+^ complex to the TALOS+ webserver (Shen et al. 2009). Figure 3 compares the predicted secondary structure for the solution complex with the secondary structure observed in the crystal form of the complex. These data are in very good agreement, which indicates that the solution conformation is very similar to the protein structure observed in crystals, and provides confidence in the assignments of the S-COMT:SAM:DNC:Mg^2+^ complex.
